# The Ribosome Biogenesis Factor Nol11 Is Required for Optimal rDNA Transcription and Craniofacial Development in *Xenopus*

**DOI:** 10.1371/journal.pgen.1005018

**Published:** 2015-03-10

**Authors:** John N. Griffin, Samuel B. Sondalle, Florencia del Viso, Susan J. Baserga, Mustafa K. Khokha

**Affiliations:** 1 Program in Vertebrate Developmental Biology, Departments of Pediatrics and Genetics, Yale University School of Medicine, New Haven, Connecticut, United States of America; 2 Departments of Genetics, Molecular Biophysics and Biochemistry, and Therapeutic Radiology, Yale University School of Medicine, New Haven, Connecticut, United States of America; Stowers Institute for Medical Research, UNITED STATES

## Abstract

The production of ribosomes is ubiquitous and fundamental to life. As such, it is surprising that defects in ribosome biogenesis underlie a growing number of symptomatically distinct inherited disorders, collectively called ribosomopathies. We previously determined that the nucleolar protein, NOL11, is essential for optimal pre-rRNA transcription and processing in human tissue culture cells. However, the role of NOL11 in the development of a multicellular organism remains unknown. Here, we reveal a critical function for NOL11 in vertebrate ribosome biogenesis and craniofacial development. *Nol11* is strongly expressed in the developing cranial neural crest (CNC) of both amphibians and mammals, and knockdown of *Xenopus nol11* results in impaired pre-rRNA transcription and processing, increased apoptosis, and abnormal development of the craniofacial cartilages. Inhibition of p53 rescues this skeletal phenotype, but not the underlying ribosome biogenesis defect, demonstrating an evolutionarily conserved control mechanism through which ribosome-impaired craniofacial cells are removed. Excessive activation of this mechanism impairs craniofacial development. Together, our findings reveal a novel requirement for Nol11 in craniofacial development, present the first frog model of a ribosomopathy, and provide further insight into the clinically important relationship between specific ribosome biogenesis proteins and craniofacial cell survival.

## Author Summary

All cells require ribosomes, the cellular factories that produce proteins. A host of factors combine to produce the multiple complex units of a ribosome, many of which are still not well understood. Surprisingly, despite the ubiquitous requirement for ribosomes, defects in various ribosome biogenesis factors cause distinct and tissue specific phenotypes, collectively known as ribosomopathies. We examined the role of one ribosome biogenesis factor, Nol11, during embryonic development to determine if it too had a tissue specific phenotype. Here we show that expression of *nol11* is strongly associated with the developing head in vertebrates and that insufficient Nol11 results in striking malformations of the craniofacial skeleton. We further show that reduced Nol11 impairs critical early steps in ribosome production, which triggers apoptosis within cell populations that contribute to the head. Increased cell death is at least partially the cause of the Nol11 craniofacial defect; reducing cell death rescues some of the craniofacial phenotype but not the ribosome biogenesis defect. In summary, we demonstrate for the first time that Nol11 is required for normal development of the vertebrate head and provide novel insight into the intriguing tissue proclivity of ribosomopathies.

## Introduction

The synthesis of ribosomes, the protein-manufacturing entities in cells, is fundamental to all of life. Therefore, it is surprising that defects in ribosome synthesis are compatible with the development of a multicellular organism, albeit with substantial morbidity. Several diseases of ribosome biogenesis, so-called ribosomopathies, have been described with a strikingly heterogeneous collection of symptomatologies [1–16]. The remarkable diversity of affected tissues across the ribosomopathies raises the question of how different cell types can be selectively affected by defects in the ubiquitous process of making ribosomes.

An emerging class of ribosomopathies includes craniofacial anomalies. Craniofacial malformations are associated with over 700 human congenital syndromes, and the molecular causes of many of these diseases remain unknown [17]. Often the structures affected in craniofacial syndromes are derivatives of the cranial neural crest (CNC), a vertebrate specific, multipotent cell population that gives rise to a vast array of craniofacial tissues, including the majority of the craniofacial skeleton [6,17–24]. Correct development of the CNC is a multistep process that includes specification at the border of the embryonic neural and non-neural ectoderm, delamination, migration to the facial primordia, proliferation and differentiation into normal craniofacial structure and morphology. This elaborate process requires extensive cellular modifications, and is carefully regulated at the molecular level [25–34]. Due to the clinical importance of the CNC, and their potential for regenerative medicine based treatment strategies, a detailed understanding of the control mechanisms underlying CNC development is a primary goal of craniofacial research.

Investigations into the surprising relationship between craniofacial disease and defects in ribosome biogenesis are beginning to emerge. Defects in CNC derived craniofacial structures are associated with the ribosomopathy known as Treacher-Collins Syndrome [6,15,16,21]. In Treacher-Collins syndrome patients, mutations in the TCOF, POLR1C or POLR1D genes result in impaired rDNA transcription and activation of the p53 mediated stress response in CNC cells [5,6,16]. Heterozygote mouse models of Treacher-Collins syndrome in certain genetic backgrounds recapitulate these craniofacial defects, which are dependent on p53 activation [6,21]. These results suggest that mechanisms regulating CNC survival may be particularly sensitive to defects in ribosome production.

Ribosome biogenesis begins with the assembly of the nucleolus around ribosomal DNA (rDNA) repeats and with the transcription of the polycistronic pre-ribosomal RNA (rRNA) precursor that contains the sequences for the 18S, 5.8S and 28S rRNAs by RNA polymerase I (RNAPI). Over 200 ribosome biogenesis factors then process these rRNAs through a complex series of cleavages and modifications to assemble into the mature small (40S) and large (60S) subunits of the ribosome by incorporating 80 ribosomal proteins and the 5S rRNA [35–38]. Maturation of the 18S rRNA is mediated by a large ribonucleoprotein called the small subunit (SSU) processome [39–41]. Previous work in yeast has revealed that members of the t-Utp/UtpA sub-complex of the SSU (Utp4, Utp5, Utp8, Utp9, Utp10, Utp15 and Utp17) are required for both optimal transcription and processing of the pre-rRNA [42]. Likewise, the orthologs of most of these proteins are also required for pre-rRNA processing and transcription in human cells. The one exception is human Utp4, which is required for pre-rRNA processing but not for RNAPI transcription [40]. Mutation of Wdr43, the zebrafish ortholog of yeast Utp5, results in p53 mediated craniofacial malformations [42,43]. Interestingly, while the majority of t-UTP/UTPA sub-complex proteins are conserved from yeast to humans, Utp8 and Utp9 constitute notable exceptions, suggesting that these t-Utp proteins may have evolved specific functions in vertebrates [40].

We recently discovered the human analog of yeast Utp8 to be NOL11, a metazoan-specific nucleolar protein [44,45]. NOL11 was discovered by its interaction with the C-terminal region of the known t-UTP/UTPA subcomplex member, hUTP4/Cirhin, the protein mutated in North American Indian childhood cirrhosis (NAIC) [4,44]. NOL11 is localized to the nucleolus, the site of ribosome biogenesis, where it associates with other proteins in the SSU processome to facilitate maturation of the small ribosomal subunit (40S). The NAIC mutation in hUTP4/Cirhin reduces interaction with NOL11, implicating NOL11 in the pathogenesis of NAIC. NOL11 is required to maintain nucleolar number and for pre-rRNA transcription and processing in human tissue culture cells but its requirement in embryogenesis remains unknown [44].

Here, we used the frog *Xenopus tropicalis* to investigate its role in vertebrate development. We report that *nol11* mRNA expression is strongly associated with the developing CNC in both amphibians and mice. We further demonstrate that *nol11* is required for optimal pre-rRNA transcription and pre-rRNA processing in *Xenopus*, and that *nol11* knockdown results in activation of the nucleolar stress response, p53 stabilization, and a dramatic craniofacial phenotype. Importantly, the craniofacial defect can be partially rescued by p53 depletion, while the underlying transcriptional defect cannot, demonstrating that the pathology is due to the cellular response to impaired ribosome biogenesis. Together, our findings reveal a novel requirement for Nol11 in craniofacial development and provide further insight into the evolutionarily conserved relationship between specific ribosome biogenesis factors and the control mechanisms that regulate CNC survival and development.

## Results

### Tissue specific expression of Nol11 during vertebrate development

**Fig 1:**
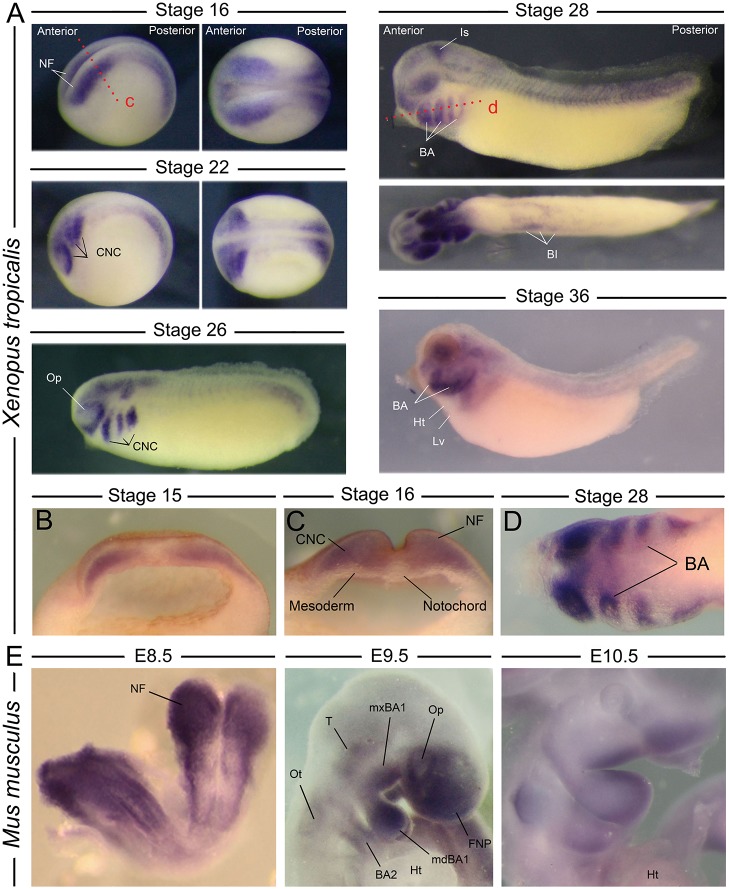
Expression of *nol11* during vertebrate development. A) Wild type *nol11* expression pattern during *Xenopus tropicalis* development. Note the strong expression in developing neural folds (NF) and the presumptive CNC at stages 16 and 22 (lateral [left] and dorsal [right] views presented). Expression is strongly associated with the migrating and differentiating CNC at subsequent stages, and is also detected in the region of the ventral blood islands (BI) and isthmus (Is) at stage 28. BA, branchial arch; Ht, heart; Lv, liver region; Op, optic placode. B) and C) Anterior transverse dissections showing expression of *nol11* in neural folds and premigratory CNC of stage 14 and 16 embryos respectively. Plane of dissection is represented by the red dotted line marked c in A. D) Horizontal dissection (shown as dotted red line marked d in A) of *nol11* expression in the branchial arches of stage 28 *Xenopus* embryos. E) *nol11* expression in E8.5, E9.5 and E10.5 wild type mouse embryos. At E8.5 expression is strongly detected in the neural folds. Transcripts are associated with CNC positive regions at both E9.5 and E10.5. BA2, 2^nd^ branchial arch; FNP, frontonasal prominence; Ht, heart; mdBA1, mandibular BA1; mxBA1, maxillary BA1; Op, optic placode; Ot, otic placode; T, trigeminal region.

As a first step in our analysis, we examined *nol11* mRNA expression during vertebrate embryonic development. Intriguingly, despite the ubiquitous need for ribosomes, *in situ* hybridization of labelled anti-sense probes revealed robust, tissue specific expression of *nol11* mRNA in both the frog and mouse. In *Xenopus*, transcripts are detected throughout the forming neural tube, and signal is particularly strong in anterior regions, from which the CNC form, at stages 14–16 (Fig. 1). This association of *nol11* expression and CNC development is maintained at later stages of development (stages 22–36), where signal marks the CNC streams as they migrate into the facial primordia and differentiate. During these stages, expression in the neural tube becomes more restricted and is detected in regions of the hindbrain, isthmus and tail. Additional stage specific expression is lightly detected in the optic and otic placodes, the ventral blood islands, and diffusely in the presumptive splenic mesenchyme (Fig. 1).

*Nol11* RNA is similarly expressed in the mouse CNC. At stage E8.5, murine *Nol11* transcripts are detected strongly in the anterior and posterior neural folds and presumptive CNC regions. At stages E9.5 and E10.5 expression is largely restricted to CNC regions, including the frontonasal prominence (FNP), the mandibular and maxillary portions of the first branchial arch (mdBA1 and mxBA1) and the more posterior BA. Interestingly, in both mice and frogs, *Nol11* expression appears enriched in distal regions of the branchial arches at later stages of development (Fig. 1). Murine expression was also observed in the optic placode, the trigeminal region and otic placode, closely mirroring the *Xenopus* expression pattern, as well as in regions of the developing gut and liver (Fig. 1, S1A Fig). Together, amphibian and murine expression suggests an evolutionary conserved relationship between the ribosome biogenesis gene *nol11* and development of the CNC.

### *Xenopus nol11* is required for normal CNC development and formation of the craniofacial skeleton

**Fig 2:**
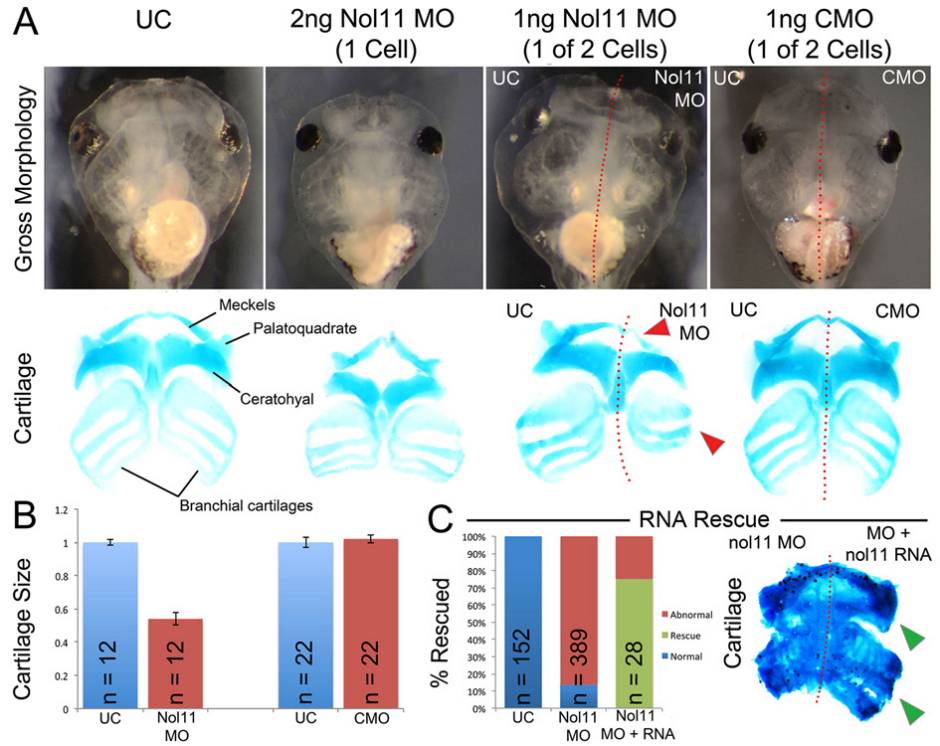
The nol11 craniofacial phenotype. A) Gross morphology and cartilage staining of UC, *nol11* whole embryo, *nol11* one-sided knockdowns and CMO one-sided knockdown embryos. Note the reduced cartilage size and abnormal morphology in *nol11* morphants (red arrowheads) while CMO injected embryos are unaffected. B) Craniofacial cartilage size is significantly reduced in *nol11* but not CMO morphants. C) Co-injection of human NOL11 RNA can rescue the cartilage phenotype in approximately 75% of treated embryos. Cartilage staining of an RNA rescued embryo; *nol11* MO was injected at the one cell stage and human *NOL11* RNA was injected into one cell at the two cell stage. Green arrowheads highlight rescued side.

To test if *nol11* is required for CNC development, we employed morpholino oligo (MO) mediated knockdown in *Xenopus tropicalis*. In *Xenopus*, MOs can be injected at the one cell stage targeting the entire embryo or at the two-cell stage where embryos targeted to either just the right or left side can be selected. To test the efficacy of our knockdown, we examined Nol11 protein levels in uninjected controls (UC) and *nol11* morphants by western blot, and confirmed a dose dependent impact on Nol11 protein levels during development (S2 Fig). Knockdown of *nol11* resulted in microcephaly and pronounced defects in CNC derived craniofacial cartilages in 86% (n = 389) of treated embryos, while control MO (CMO) injected embryos were unaltered (Fig. 2A and 2C). Cartilaginous defects included significantly reduced size and dysmorphology of the Meckel’s, quadrate, ceratohyal and branchial cartilages at stage 45, (Fig. 2A and 2B). The severity of these defects varied from a 27% to 55% reduction in cartilage size, with an average reduction of 42% (Fig. 2B). These cartilages are derivatives of distinct branchial arches (BAs) and neural crest migratory streams, and their loss demonstrates a central role for *nol11* in CNC development, independent of axial level of origin. *nol11* morphants did not survive beyond stage 45–47, probably due to a non-functional jaw apparatus and a reduced ability to feed. To test the specificity of the *nol11* MO, we injected human *NOL11* mRNA into one cell of two cell stage *nol11* morphants and compared the two sides. *NOL11* mRNA rescued both cartilage size and morphology in 74% of embryos (Fig. 2C).

**Fig 3:**
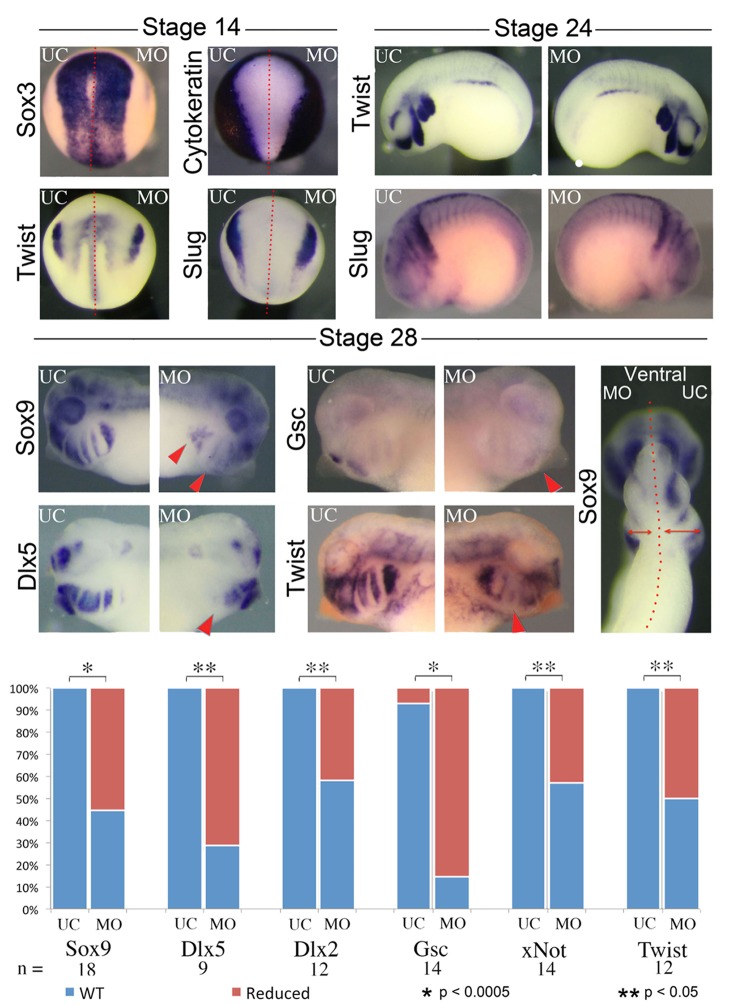
Knockdown of *nol11* disrupts cranial neural crest development. At stages 14 and 24 neural and CNC development appears normal in one side treated embryos, as assayed by expression of key marker genes including *sox3*, *cytokeratin*, *twist* and *slug*. By stage 28 however, reductions are apparent in the expression of numerous CNC genes. The branchial arches are also smaller on the MO treated side at stage 28. Graph displays number of embryos exhibiting abnormal gene expression in control and *nol11* morphant embryos.

To probe the ontogeny of these cartilaginous defects, we next examined development of the CNC in *nol11* morphants. We found that initial specification of dorsal territories and neural tissue proceeds normally in *nol11* morphants, as assayed by expression of the pan-neural marker *sox3*, the exclusion of cytokeratin expression from neural tissue, and expression of *pax2* and myod in the developing neural tube and somites respectively at stage 14/15 [46–48] (Fig. 3, S3 Fig). With the exception of a slight delay, expression of the CNC master genes *twist*, *slug*, *ap2* and *sox9* [49–51] also appeared normal relative to controls at stages 14 and 24, suggesting that induction, delamination and migration of the CNC occurs in a largely normal fashion following *nol11* knockdown (Fig. 3, S3A Fig). However, by stage 28, defects in CNC markers are readily apparent within the developing BAs. These include striking reductions in *sox9* expression (a CNC gene required for cartilage differentiation), as well as CNC patterning genes including *gsc*, *dlx5*, *twist*, *dlx2* and *xnot* (Fig. 3, S3 Fig). Importantly, the branchial arches were also observed to be smaller on the treated side of the embryo, suggesting a reduced number of CNC cells (Fig. 3, S3 Fig). While the medio-lateral size of the neural tube appeared slightly reduced in morphants at stage 28, patterning of the neural tube and brain appeared grossly intact, as assayed by expression of *pax2*, *hoxb3*, and *sox3* (S3B Fig).

To test if patterning defects were selective for the CNC, we assayed various markers for different organs testing cardiac, gut and kidney development. Both cardiac looping and *pitx2* expression were normal in morphants (S1B, D Fig), demonstrating that the strong *nol11* expression observed in the embryonic left-right organizer is not required for normal organ *situs* [52]. Gut morphology was abnormal in morphants at stage 45. We also examined expression of *nkx2*.*5*, *pax2*, *sglt1*, *smp30* and *hex* in stage 28–36 *nol11* morphants but found no marked changes (S1C, E, F Fig). Interestingly while expression of the cardiac and splenic patterning gene *nkx2*.*5* [53,54] appeared normally positioned in morphants, its splenic expression appeared moderately reduced, suggesting a possible role for *nol11* in spleen development (S1C Fig). In future studies, it will be interesting to examine late stage development of additional organs.

### The Nol11 craniofacial defect is associated with increased apoptosis

**Fig 4:**
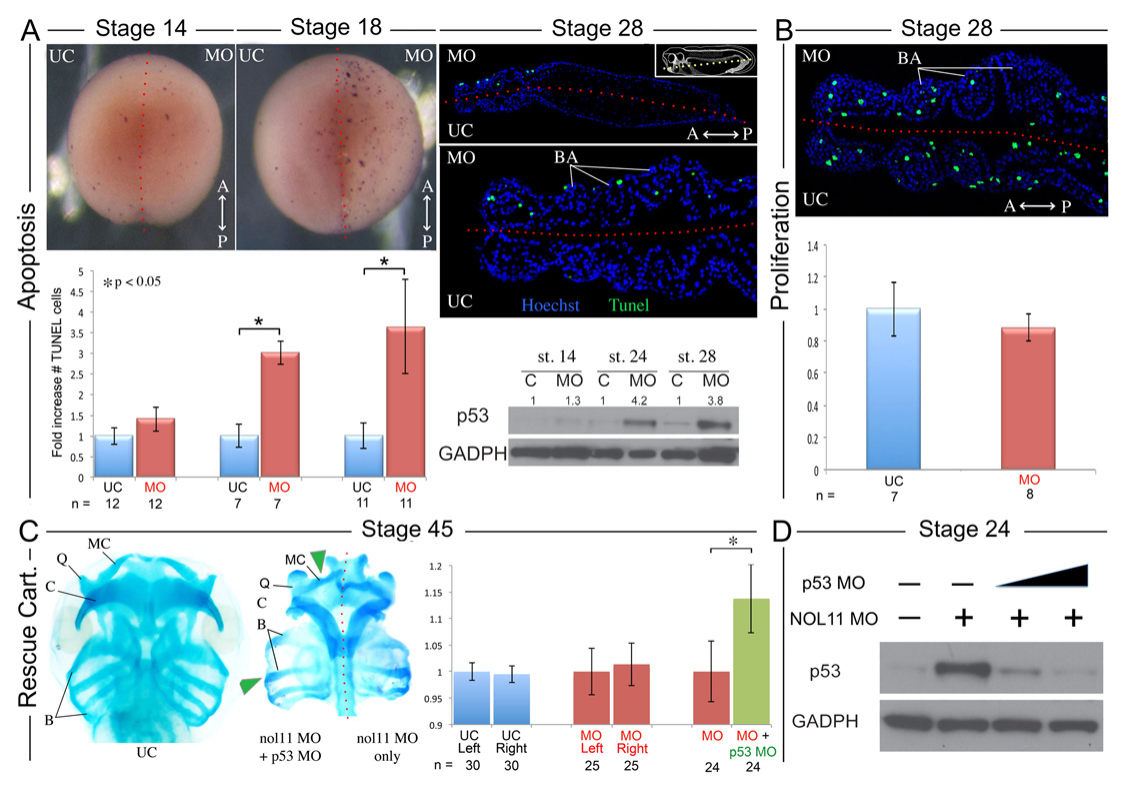
Increased apoptosis underlies the *nol11* cartilage defects. A) *nol11* knockdown results in a progressive increase in apoptosis. At stage 14 no significant difference was observed in rates of TUNEL staining between knockdown and control halves of the embryo. At stages 18 and 28 increased apoptosis was evident on the treated side of whole mount and sectioned paraffin embedded embryos. Note that this increased apoptosis occurs primarily within the craniofacial ectomesenchyme. The graph represents the relative quantification of apoptosis rates at stages 14, 18 and 28. This stage specific increase in apoptosis was confirmed by a similar increase in p53 protein levels in 1 cell injected embryos as assayed by western blot (lower right panel). Dotted red lines mark the embryonic midline. B) No significant change in proliferation rates was noted following *nol11* knockdown. C) Inhibition of apoptosis by p53 MO results in a partial rescue of cartilage size and morphology. Each pair of columns in the graph compares cartilage size measured in bilateral halves of embryos. The blue pair reveals no significant difference in cartilage measurements in the left vs right side of the UC embryonic head. In the second pair (red), cartilage size is seen to be comparable on either side of the *nol11* morphant head. The final pair illustrates that cartilage size is significantly improved on the side of *nol11* morphants rescued with p53 MO (green) relative to the side that received *nol11* MO only (red). D) Western blot demonstrating that the p53 MO efficiently reduces p53 protein levels in *nol11* morphants.

**Fig 5:**
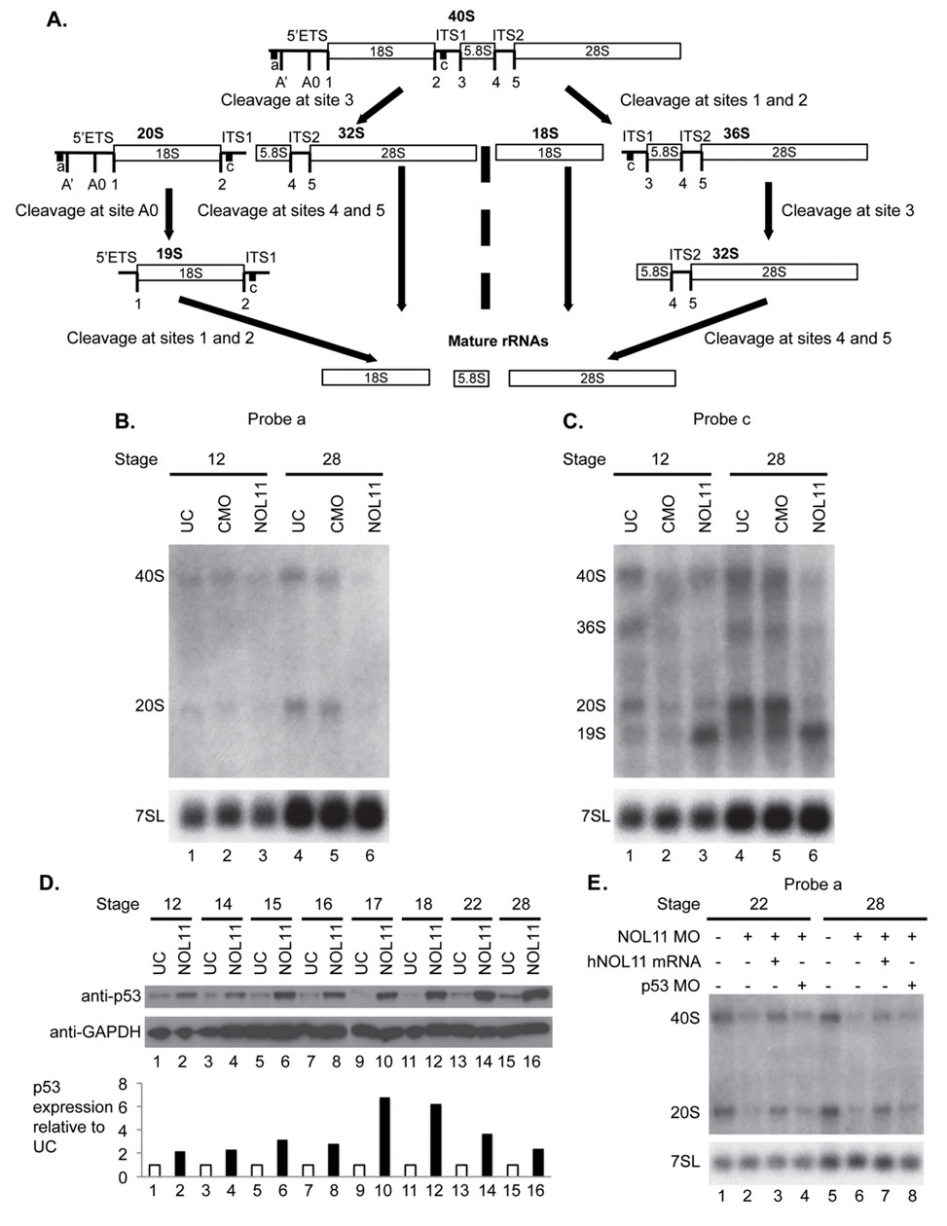
Nol11 depletion impairs rDNA transcription and pre-rRNA processing in *X*. *tropicalis*. A) Scheme of pre-rRNA processing pathways in *X tropicalis*. The pre-rRNA is transcribed by RNAPI as a 40S polycistronic precursor. Several cleavages are required to separate the mature rRNAs. The locations of oligonucleotide probes used for northern blots are indicated by lettered lines (a, c) and the cleavage sites indicated. This scheme was adapted from [71–75]. B) Morpholino (MO) depletion of Nol11 impairs pre-rRNA transcription at stage 28. The northern blot was hybridized with probe a (Fig. 5A) and with a probe to the 7SL RNA as a loading control (lower panel). Bands were quantified and analysed by RAMP ([60]; S6A,B Fig) C) Morpholino (MO) depletion of Nol11 impairs pre-rRNA transcription and processing. The northern blot was hybridized with probe c (Fig. 5A) and with a probe to the 7SL RNA as a loading control (lower panel). Bands were quantified and analysed by RAMP ([60]; S6C,D, E, F Fig). D) Depletion of Nol11 leads to increased p53 levels. The expression of p53 from control and *nol11* depleted embryos was analysed by western blot with anti-p53 antibodies. GAPDH levels were used as a loading control. Values for p53 expression normalized to GAPDH are represented in the bar graph. E) MO-resistant human NOL11 (hNOL11) mRNA but not p53 depletion rescues pre-rRNA levels. Embryos injected as shown by + and—in the figure at stages 22 and 28. The pre-rRNAs were visualized with probe a on a northern blot; hybridization to the 7SL RNA was used as a loading control.

As mutations in ribosomal biogenesis factors have previously been associated with altered cell survival and since the reduced size of the BAs in *nol11* morphants suggests reduced cell numbers, we next examined rates of apoptosis and proliferation in the facial primordia of *nol11* depleted embryos. At early stages of development, no significant change was observed in apoptosis rates as assayed by TUNEL staining and p53 protein levels (Fig. 4A). However by stages 18 through 28, as the CNC migrate into and become patterned within the facial primordia, we observed a dramatic 3–4 fold increase in the number of TUNEL stained cells (Fig. 4A, S4 Fig). This increase correlates well with the observed onset of the cartilage phenotype. Examination of TUNEL staining in paraffin sections revealed the increased apoptosis to be largely confined to ectomesenchymal cells located with the facial primordia, possibly CNC (Fig. 4A). Western blot analysis of p53 protein levels in morphants revealed approximately normal protein levels at early time points (st12–14) but a progressive increase over subsequent stages, culminating in an approximately 4-fold increase between stages 18–28. These findings mirror the TUNEL results and confirm a stage dependent increase in apoptosis (Fig. 4A, Fig. 5D). Interestingly, in contrast to observations in the zebrafish Wdr43 mutant, nol11 morphants did not have a significant change in rates of proliferation (Fig. 4B).

In order to confirm that this increase in apoptosis contributes to the *nol11* craniofacial phenotype, we next asked if knockdown of p53 could rescue the malformation. Interestingly, injection of p53 MO alone into wild type *Xenopus* embryos produces a dose dependent craniofacial defect, most plausibly due to disruption of normal patterns of cell death during development or, as previously reported, apoptosis independent effects [55,56]. However, we found that by titrating the p53 knockdown we could ameloriate some of the *nol11* cartilage phenotype, including improved cartilage size and morphology (Fig. 4C). The efficacy of the p53 MO in reducing p53 protein levels was demonstrated by western blot (Fig. 4D). We also found that injection of p53 MO dramatically reduced rates of cell death within the branchial arches of stage 28 *nol11* morphants, while CMO had no effect (S4 Fig). Together these findings suggest that the *nol11* craniofacial phenotype is at least in part caused by an increase in p53 mediated cell death within the cells that populate the facial primordia.

### Nol11 is required for ribosome biogenesis in a developing embryo

Increased tissue specific apoptosis is associated with disruption of several ribosome biogenesis factors, including Tcof1, the protein that is mutated in Treacher-Collins syndrome [1,6,21,43,57]. Under conditions of defective ribosome biogenesis, this apoptotic response is triggered by the ‘nucleolar stress response’, the binding of excess ribosomal proteins, such as RPL5 and RPL11, to Mdm2, which in turn prevents Mdm2 mediated p53 degradation [58]. With this in mind, we sought to investigate whether pre-rRNA processing and transcription, and thus ribosome biogenesis are disrupted in *nol11* morphants.

To develop probes for northern blots to examine ribosome biogenesis in *X*. *tropicalis*, we first identified the *X*. *tropicalis* 5’ external transcribed spacer (ETS) in the pre-rRNA using a BLAST search. Aligning *X*. *laevis* and *X*. *borealis* pre-rRNA sequences, we identified two *X*. *tropicalis* Xentr7.1 scaffolds (xenbase.org). Scaffold sequence 169 contained sequences similar to *X*. *laevis* 5’ETS, 18S rRNA, internal transcribed spacer 1 (ITS1), and the 5’ portion of the 5.8S rRNA while scaffold 2385 only contained sequence similar to the 5’ portion of the 5’ETS (S5 Fig). Using these alignments we designed oligonucleotides that were reverse complements of sequences in the 5’ETS and ITS1 of *X*. *tropicalis* since NOL11 is required for biogenesis of the mature 18S rRNA in human cells [44]

Using these oligonucleotide probes (probes a,c indicated in Fig. 5A), we investigated the effect of *nol11* knockdown on ribosome biogenesis in *X*. *tropicalis* embryos by northern blot analysis (Fig. 5B and 5C). Hybridization to the 7SL SRP RNA was carried out as a loading control, as done previously [59]. Northern blots were repeated 3 times with the indicated embryos. The results were quantified and studied by Ratio Analysis of Multiple Precursors (RAMP; S5 Fig; [60]). With either probe at either stage 12 or stage 28, no significant difference was observed either by eye (Fig. 5B and 5C) or in the RAMP profile between uninjected controls (UC) and control morpholino injected embryos (CMO) (S6 Fig), indicating that injection *per se* does not result in defects in ribosome biogenesis as detected by northern blots.

We do, however, observe defects in transcription and pre-rRNA processing in *nol11* morphants, indicating that depletion of Nol11 results in defective ribosome biogenesis. In northern blot analysis with probe a, steady state levels of both the 40S pre-rRNA primary transcript and its 20S pre-rRNA processed product were reduced in stage 28 *nol11* morphants (Fig. 5B, lanes 4–6; S6B Fig). In contrast, no significant difference was detected between *nol11* morphants and the controls at stage 12 (Fig. 5B, lane 1–3; S6A Fig). The marked decrease in steady state levels of both pre-rRNA transcripts in *nol11* MO-treated embryos at stage 28 is consistent with a defect in pre-rRNA transcription following Nol11 knockdown.

Likewise, northern blot analysis with probe c revealed defects in transcription and pre-rRNA processing in *nol11* MO-treated embryos. When the levels of the indicated pre-rRNAs were compared to the 7SL RNA loading control in *nol11* morphants, a significant decrease in the 40S, 36S and 20S pre-rRNAs was detected at stage 28 (Fig. 5C, lanes 4–6; S6D Fig). This is consistent with a defect in pre-rRNA transcription. At the same time, the 19S pre-rRNA (Fig. 5A) accumulated in *nol11* MO-treated embryos at stage 28 (Fig. 5C, lanes 4–6; S6D Fig), consistent with a defect in pre-RNA processing. No significant difference was detected between *nol11* morphants and the controls at stage 12 when the pre-rRNA levels were compared to the 7SL RNA loading control (Fig. 5C, lane 1–3; S6C Fig). However, when the levels of the indicated pre-rRNAs were compared to each other in *nol11* morphants, accumulation of the 19S pre-rRNA could be observed as early as stage 12 (Fig. 5C, lane 3; S6E Fig) and was very pronounced by stage 28 (Fig. 5C, lane 6; S6F Fig). Thus, depletion of Nol11 by injection of MO in developing *X*. *tropicalis* embryos results in defective pre-rRNA transcription and processing.

As discussed above, defects in ribosome biogenesis can lead to p53 stabilization and subsequent apoptosis due to a cellular phenomenon known as nucleolar stress [61–63]. In order to further examine the relationship between the pre-RNA transcription defect and increased p53 expression, we compared changes in both over the relevant embryonic time course. As described above, MO-depletion of *nol11* caused an increasingly severe disruption of pre-RNA transcription over these developmental stages (Fig. 5B and 5C, lane 6; S6B,D Fig). We found that this correlated precisely with the progressive increase in p53 levels by western blot (Fig. 5D). Together, these findings indicate that *nol11* depletion results in reduced pre-RNA transcription, impaired ribosome biogenesis and increased levels of p53, a hallmark of nucleolar stress.

To confirm that these defects are specific to *nol11* depletion, we compared *nol11* morphants with morphants rescued with NOL11 mRNA. Co-injection of the human NOL11 mRNA rescued the steady state levels of the pre-rRNAs in morphants (Fig. 5E, lanes 3 and 7) indicating restoration of pre-rRNA transcription and therefore ribosome biogenesis. In addition, co-depletion of p53 and Nol11 did not rescue the pre-rRNA transcription defect although we did find a partial rescue of the craniofacial phenotype (Fig. 5E, lanes 4, 8 and Fig. 4C, above).

## Discussion

Ribosome biogenesis is a vital and energy intensive process, requiring the interaction of hundreds of proteins and the majority of a cell’s transcriptional output. As such, it is not surprising that it is carefully monitored, or that a control mechanism such as the nucleolar stress response exists to remove compromised cells. Indeed, impaired ribosome production is frequently associated with apoptosis. For example, Treacher-Collins syndrome is molecularly characterized by impaired ribosome biogenesis, activation of the nucleolar stress response and increased p53 levels in CNC cells [6,21]. A similar increase in apoptosis has been reported to underlie the zebrafish Wdr43 (Utp5) craniofacial phenotype [43]. Importantly, the observation that inhibition of apoptosis can rescue the cartilage phenotype but not the underlying transcriptional and processing defect in both *Xenopus nol11* morphants and some mouse Treacher-Collins Syndrome models demonstrates that it is the evolutionary conserved nucleolar stress response, and not the ribosomal defect *per se*, that produces the craniofacial malformation. As such, further investigation and modulation of this stress response may ultimately provide novel treatment options for apoptosis induced ribosomopathies. Together with previous studies, our data provide further evidence that the CNC are particularly sensitive to insufficiency of certain ribosome biogenesis proteins and reveal a common, evolutionarily conserved control mechanism through which compromised multipotent CNC are eliminated in the embryo. This sensitivity of CNC to ribosome biogenesis defects also suggests that mutations in nucleolar factors may contribute to additional currently unexplained human congenital defects.

The finding that defects in presumably globally required ribosome biogenesis factors can produce tissue specific defects is an intriguing emerging facet of embryonic development. Several human diseases are now known to result from mutations in ribosome production and present with distinct phenotypes [1–4,6,8,9,11,14,16,44,64,65]. The mechanisms underlying the tissue specificity of these ribosome biogenesis factors remain unknown but may include differential ribosomal biogenesis factor requirements during the development of distinct tissues or a heightened sensitivity to impaired protein production in particular cell populations. While the CNC is a highly active embryonic cell population, the distinct phenotypes observed in human ribosomopathies, many of which do not include craniofacial defects, would seem to argue against the latter scenario. Furthermore, experimental support for the differential requirements hypothesis is beginning to emerge from studies of both mice and zebrafish [6,43,57,66]. Our findings that *nol11* expression at the mRNA level is tightly linked to CNC cells, where it is required for normal differentiation and development of craniofacial cartilages, but not detected in, or associated with overt defects in the development of several other active cell populations at similar stages, provides strong support for the possibility of differential ribosomal protein requirements during embryonic development. While beyond the scope of this work, a future comparison of RNA translation and protein profiles between the CNC and non-CNC cells of wild-type and knockdown embryos would be highly informative.

Importantly, the role of the NOL11 protein in ribosome biogenesis is conserved between human cells and *X*. *tropicalis* embryos. In HeLa cells [44] and in developing frog embryos, depletion of NOL11 results in defects in pre-rRNA transcription and pre-18S rRNA processing. However, the precise pattern of the pre-rRNAs differs between the two species even in the non-depleted state, with the pattern in *X*. *tropicalis* similar to that found in zebrafish (*D*. *rerio*) [43,67]. Whether this is due to differences among vertebrate species or differences between tissue culture cells and a developing organism we do not yet know. The development of oligonucleotide probes to study these pre-rRNA pathways in *X*. *tropicalis* should thus facilitate the mapping of steps in pre-rRNA processing in this developing frog.

In summary, we have demonstrated a novel role for *nol11* in vertebrate ribosome biogenesis, cell survival and craniofacial development. The emerging relationship between specific ribosome biogenesis factors and tissue specific developmental defects is an unexpected and intriguing feature of developmental biology, and one that may underlie numerous currently unexplained congenital syndromes. Our findings provide insight into this relationship and highlight the utility of *Xenopus tropicalis* as a model for further investigations of human ribosomopathies.

## Materials and Methods

### Ethics statement

*X*. *tropicalis* were maintained and cared for in our aquatics facility, in accordance with Yale University Institutional Animal Care and Use Committee protocols.

### Embryos

Embryos were produced by *in vitro* fertilization and raised to appropriate stages in 1/9MR + gentamycin. Fixed wild-type mouse embryos were obtained from C. Wilson and C. Bogue.

### Antisense morpholino knockdown

Antisense morpholino oligonucleotides targeting the *nol11* translational start site (5’ GCTCCCCGAGAGCGGCCATCTTGTC 3’), or standard CMO from Genetools LLC, Philomath, OR) were injected at either the one cell stage (2ng MO) or into one cell at the two-cell stage (1ng MO). A full-length cDNA clone of human NOL11 was purchased from Open Biosystems and subcloned into the Gateway Entry vector pDONR221 (Invitrogen). The specificity of the MO was then tested by injecting 2ng of *nol11* MO at the one cell stage and subsequently injecting 50pg of human NOL11 RNA into one cell at the two cell stage in order to rescue the phenotype on the RNA treated side. Rescue was scored by increased cartilage size and morphology.

### Whole-mount in situ hybridization

*Xenopus nol11* template for probe was amplified from wild type cDNA using the following primer combinations: forward: 5’- AGTTTGGTGAGGCGCTGTAT-3’; reverse: 5’- GGCTCATCCAATCCACTACC-3’ and then TOPO TA cloned (Life Technologies) as per manufacturer’s instructions. Digoxigenin-labeled antisense probes for *ap-2*, TGas030K20; *cytokeratin*, IMAGE:6991625; *dlx2*, IMAGE:6980076; *dlx5*, TNeu071c08; *gsc*, TGas129E16; *hex*, TGas075h17; *myoD*, TNeu017H11; *nkx2*.*5*, IMAGE:7517699; *nol11*, as described above; *pax2*, TNeu062i10; *pitx2*, TNeu083k20; *sglt*, IMAGE:5308256; *sox9*, TNeu111f21; *slug*, TNeu008A21; *smp-30*, IMAGE:6999181; *twist*, TNeu125e01 and *xnot*, TNeu017e01 were *in vitro* transcribed with T7 High Yield RNA Synthesis Kit (E2040S) from New England Biolabs. Embryos were collected at the desired stages, fixed in MEMFA for 1–2 hours at room temperature and dehydrated into 100% ETOH. Whole mount *in situ* hybridization was performed as described previously [68]. Embryos were stained with BM Purple and examined after equilibration in 100% glycerol.

### Alcian Blue staining and measurements of cartilage

Stage 45 embryos were fixed in MEMFA for 20 mins at RT and then washed in acid alcohol (1.2% HCL in 70% ETOH) before staining in 0.5% alcian blue solution in acid alcohol over night at 4 degrees. Samples were then washed several times in acid alcohol and dehydrated into H_2_O, before bleaching for 1–2 hrs in 1.2% hydrogen peroxide. Samples were then washed several times in 2% KOH and left rocking overnight in 10% glycerol in 2% KOH. They were then processed through 20%, 40%, 60% and 80% glycerol in 2% KOH. The facial cartilages were then dissected out and imaged using a Canon EOS 5d digital camera mounted on a Zeiss discovery V8 stereomicroscope. Bilateral cartilages were then outlined using ImageJ software (NIH) and their relative sizes measured.

### Western blotting

Pools of ten staged control and morphant embryos were collected and placed in 100ul of 1 x RIPA buffer. Embryos were then crushed using a pestle and spun down twice to separate protein from fat and debris. Western blots were then carried out following standard protocols, using an anti-p53 (Thermo Scientific, MA1–12549 1:800 dilution) or an anti-NOL11 (SIGMA HPA022010 1:1000 dilution) primary antibody and an anti-mouse or anti-rabbit HRP conjugated secondary (Jackson Immuno Research Laboratories, 715–035–150 or 211–032–171 1:15000 dilution). Anti-GAPDH (Ambion, AM4300 1:5000 dilution) was used as an internal control. Quantifications of changes in protein level were calculated using ImageJ software from NIH.

### TUNEL and proliferation assays

Whole mount TUNEL staining of developmentally staged wild-type and nol11 morphant embryos was carried out as previously described [69]. Stage 28 embryos were also fixed in 4% paraformaldehyde, embedded in paraffin and cut into 10 μm sections. The TUNEL assay was carried out using the *In situ* cell death detection kit, Fluorescein (Roche Applied Science, 11684795910) as per the manufactures instructions (using the trypsin pretreatment option described). Cell proliferation assays were carried out using an anti-phospho-Histone H3 antibody (Millipore, 06–570) and an Alexa Fluor 488 Chicken Anti-Rabbit (Life Technologies, A21441).

### Apoptosis rescue

In order to determine if the *nol11* phenotype is due to increased apoptosis, we injected 2ng of *nol11* MO at the one cell stage. A subset of these embryos were then injected with 1ng of *p53* MO into one cell at the two cell stage to rescue the phenotype on the p53 MO treated side. Rescue was scored by measuring and comparing the area of cartilage present on the *nol11* MO only vs the *nol11* + *p53* MO treated sides of the midline using ImageJ software from NIH. Similar comparisons were made between the left and right sides of uninjected and *nol11* MO only embryos as controls.

### Statistical analyses

Each experiment was performed a minimum of three times. Statistical significance of the frequency of CNC patterning gene disruptions was tested with a Chi-squared test. The significance of TUNEL rates, changes in cartilage size and rescue experiments was evaluated using paired or unpaired, two-tailed student’s *t*- tests as appropriate.

### Northern blotting

*X*. *tropicalis* embryos were injected with MOs and mRNA as described. RNA was harvested from embryos by dissolving 10–15 embryos in TRIzol (Invitrogen) and total RNA was extracted per the manufacturer’s instructions. Northern blot analyses were performed as described previously [70]. Two or four μg of total RNA per sample were separated by gel electrophoresis on a 1% agarose/1.25% formaldehyde gel and then transferred to a nylon membrane (Hybond-XL, GE Healthcare). RNA species were detected by hybridization with radiolabelled oligonucleotide probes or by methylene blue staining. Oligonucleotide probes are as follows: Probe a, 5’-CAC TAA GGG TCA ACC TCT CCT T-3’; Probe c, 5’-CAG GTA CCC GGG TCG GCC TGC GGC G-3'; 7SL: 5’- CAT ATT GAT ACC GAA CTT AGT GC-3’.

Northern blots were quantitated using a phosphorimager (Bio-Rad Personal Molecular Imager) and the levels of all RNAs were normalized to the uninjected control. Ratio Analysis of Multiple Precursors (RAMP) was performed as in [60]. Statistical analysis was performed using a 2-way ANOVA with Tukey’s multiple comparisons test for post hoc analysis of significance in GraphPad Prism.

## Supporting Information

S1 Fig *nol11* in vertebrate development. A) Whole mount in situ hybridization of digoxigenin labelled *Nol11* probe in E9.5 and E10.5 mouse embryos (E10.5 sample shown has been hemisected). BA, branchial arch; FNP, frontonasal prominence; Ht, heart; mdBA1, mandibular BA1; mxBA1, maxillary BA1; Op, optic placode; Ot, otic placode. B) Gross morphology of stage 45 *nol11* morphants. Gut morphology is abnormal in morphants, while organ *situs* appears largely normal relative to wild type controls. Heart (red), gall bladder (green) and gut (yellow) are pseudocoloured in lower right panels. C) Left sided expression *nkx2*.*5* is reduced or absent in the splenic anlage of a subset of *nol11* morphants (compare red arrowheads). D) Example of the normal sided *pitx2* expression present in *nol11* knocked down embryos. E) Kidney development appears largely intact in *nol11* MO treated side compared to control side. F) Quantification of number of embryos displaying the described phenotypes.
(TIF)

S2 Fig Efficacy of the *nol11* MO. A) Western blot demonstrating that injection of *nol11* MO at the one cell stage reduces Nol11 protein levels at stage 28 in a dose dependent manner. C, control, 2, 2ng nol11 MO, 3, 3ng nol11 MO. B) Injection of 2ng of *nol11* MO at the one cell stage results in a 30% reduction of protein level at stage 15 and a 44% reduction at stage 23.
(TIF)

S3 Fig Patterning and Apoptosis in *nol11* morphants. A) Somite, CNC and neural development appear intact in *nol11* morphants at stage 14 as assayed by *myoD*, *ap2* and *pax2* expression. Expression of *xnot* appears reduced at stage 28. B) Reduced *dlx5* expression and BA hypoplasia on treated side of a stage 28 embryo. Neural patterning is normal at this stage as assayed by expression of *pax2*, *hoxb3* and *sox3*. C) Whole mount TUNEL staining of treated and untreated sides of a stage 28 embryo. Note the increased staining in the craniofacial regions of the morphant side.
(TIF)

S4 Fig Rates of cell death in the branchial arches. A) Graph of the number of Tunel positive cells per 100 cells present in sections of the branchial arch region of stage 28 UC, CMO, Nol11 MO, Nol11 MO + p53 MO, and p53 MO only injected embryos (* = P < 0.05). B) Whole mount stage 28 embryo with plane of section represented by the red dotted line. Representative Tunel stained (green) sections from each control and morphants.
(TIF)

S5 Fig The *X*. *tropicalis* 5’ external transcribed spacer. The 5’ETS sequences for *X*. *laevis* (GeneBank ID: X02995.1; nucleotides 318–1029) and *X*. *borealis* (GeneBank ID: X00184.1; nucleotides 545–1156) were aligned to *X*. *tropicalis* scaffolds 169 and 2385 from the Xentr7.1 database.
(TIF)

S6 Fig Statistical analysis of pre-rRNA levels by Ratio Analysis of Multiple Precursors (RAMP) [60]. RNAs were quantitated from northern blots using a phosphorimager (Bio-Rad Personal Molecular Imager). All ratios are representative of three biological replicates (n = 3). The levels of all RNAs were normalized to the uninjected control (UC). All statistical analyses for significance for Nol11-depleted embryos (NOL11) were performed compared to the control morpholino injected embryos (CMO). A. Pre-rRNA levels in Nol11-depleted embryos are not significantly affected at stage 12 compared to CMO as shown by a probe in the 5’ETS (probe a). B. At stage 28, the levels of 40S and 20S pre-rRNAs, are significantly decreased relative to the loading control 7SL RNA for Nol11-depleted embryos compared to CMO as shown by probe a. This is consistent with decreased pre-rRNA transcription. C. Pre-rRNA levels relative to the 7SL RNA for Nol11-depleted embryos are not significantly affected compared to CMO at stage 12 as shown by a probe in the ITS1 (probe c). D. At stage 28 for Nol11-depleted embryos, the 40S, 36S, and 20S pre-rRNAs are all significantly decreased relative to the 7SL RNA compared to CMO as shown by probe c. This is consistent with decreased pre-rRNA transcription. However, the levels of 19S are significantly increased relative to the 7SL RNA compared to CMO as this precursor accumulates in Nol11-depleted embryos. This is indicative of a pre-rRNA processing defect. E. and F. For both stage 12 and 28 Nol11-depleted embryos, the ratios of 19S/40S, 19S/36S, and 19S/20S are significantly increased compared to CMO indicating accumulation of the 19S pre-rRNA relative to the other three pre-rRNAs that hybridize with probe c. This is indicative of a pre-rRNA processing defect. [ns = p>0.05 (not significant), * = p≤0.05, ** = p≤0.01, *** = p≤0.001, **** = p≤0.0001]
(TIF)
