# Supplementary figures and images for: The Ribosome Biogenesis Factor Nol11 Is Required for Optimal rDNA Transcription and Craniofacial Development in Xenopus
Source: PLoS Genet. 2015 Mar 10;11(3):e1005018. doi: 10.1371/journal.pgen.1005018 (PMC4354908; doi:10.1371/journal.pgen.1005018)

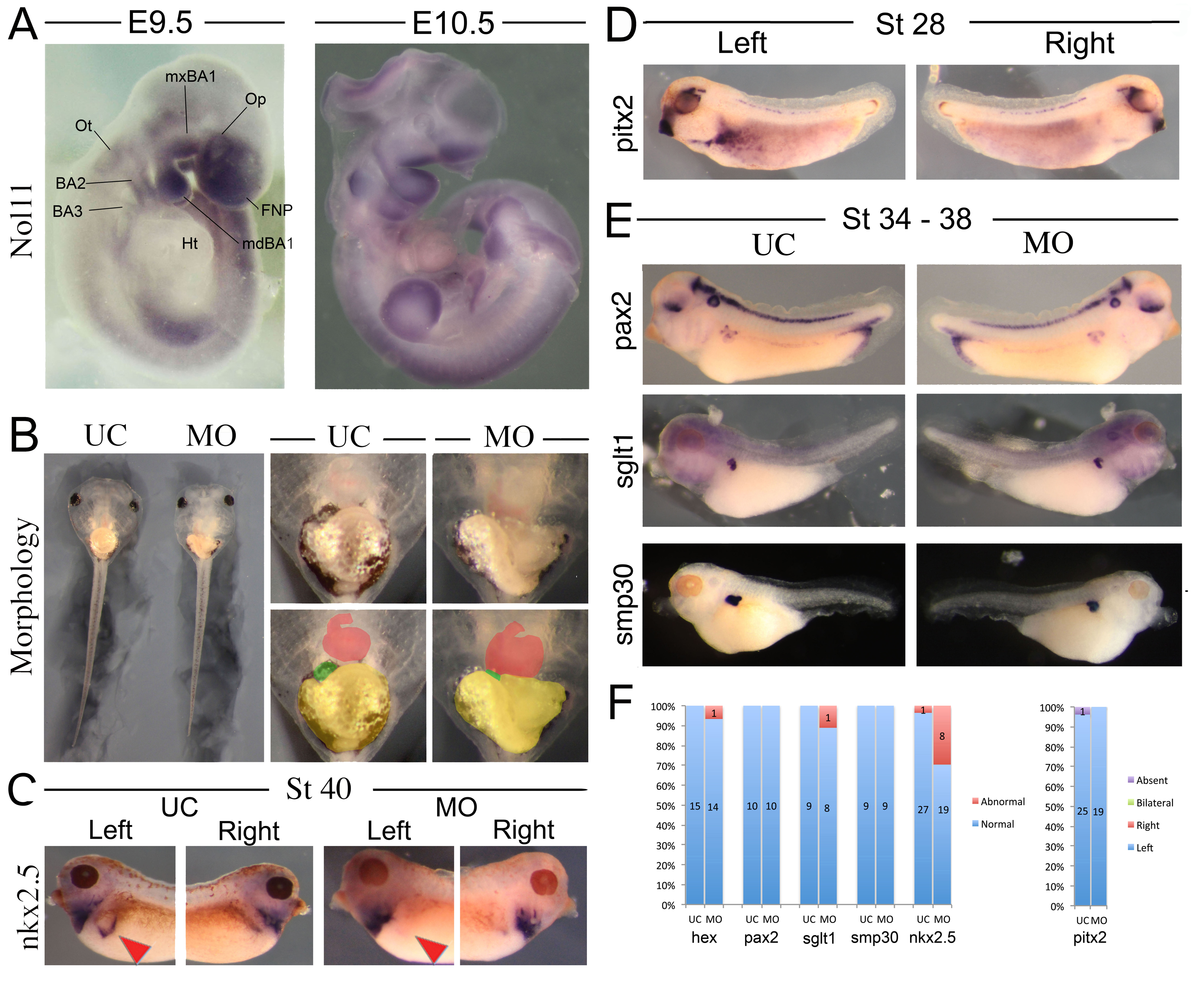

Supplement: S1 Fig — A) Whole mount in situ hybridization of digoxigenin labelled Nol11 probe in E9.5 and E10.5 mouse embryos (E10.5 sample shown has been hemisected). BA, branchial arch; FNP, frontonasal prominence; Ht, heart; mdBA1, mandibular BA1; mxBA1, maxillary BA1; Op, optic placode; Ot, otic placode. B) Gross morphology of stage 45 nol11 morphants. Gut morphology is abnormal in morphants, while organ situs appears largely normal relative to wild type controls. Heart (red), gall bladder (green) and gut (yellow) are pseudocoloured in lower right panels. C) Left sided expression nkx2.5 is reduced or absent in the splenic anlage of a subset of nol11 morphants (compare red arrowheads). D) Example of the normal sided pitx2 expression present in nol11 knocked down embryos. E) Kidney development appears largely intact in nol11 MO treated side compared to control side. F) Quantification of number of embryos displaying the described phenotypes. (TIF) [file pgen.1005018.s001.tif]

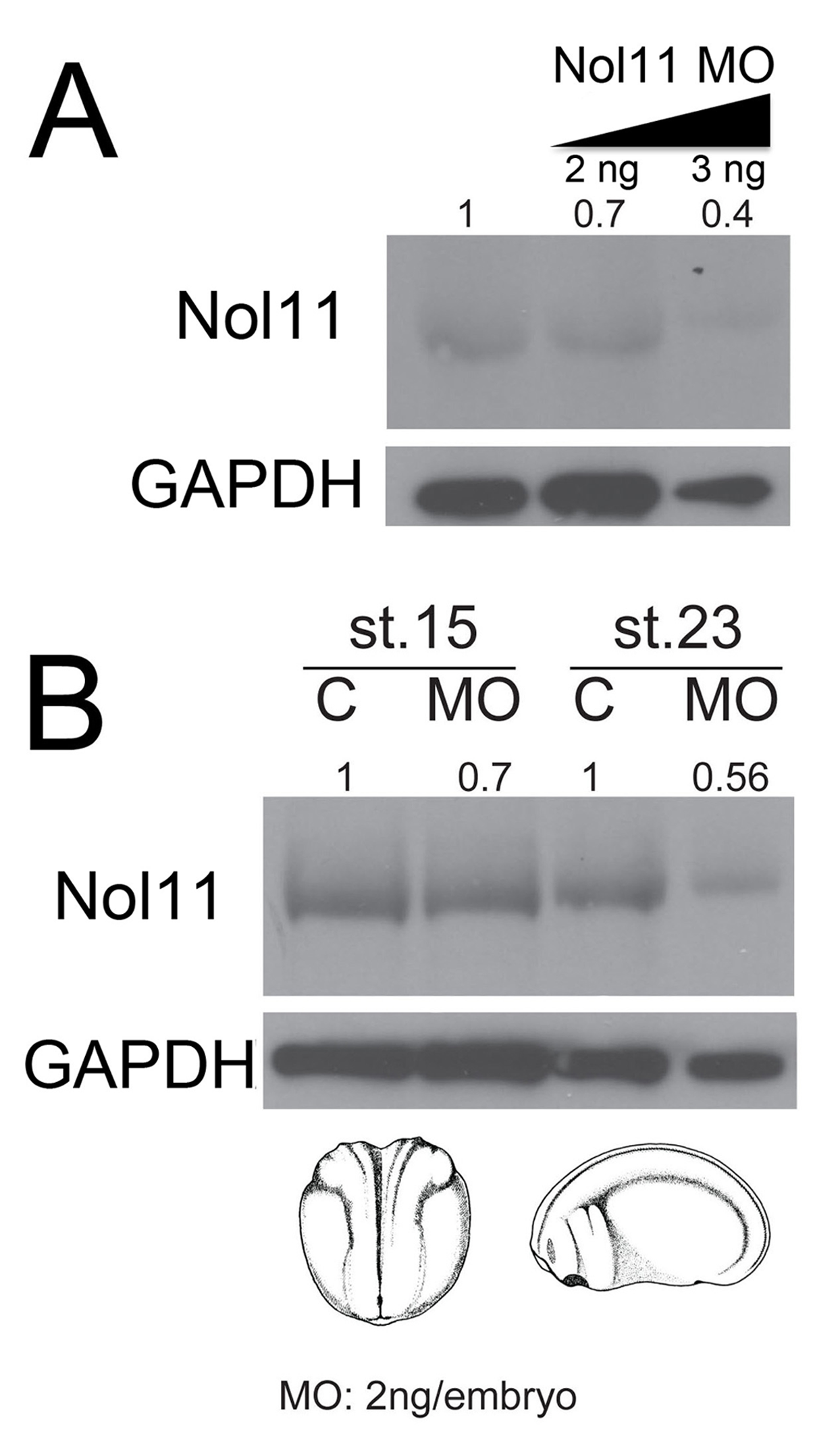

Supplement: S2 Fig — A) Western blot demonstrating that injection of nol11 MO at the one cell stage reduces Nol11 protein levels at stage 28 in a dose dependent manner. C, control, 2, 2ng nol11 MO, 3, 3ng nol11 MO. B) Injection of 2ng of nol11 MO at the one cell stage results in a 30% reduction of protein level at stage 15 and a 44% reduction at stage 23. (TIF) [file pgen.1005018.s002.tif]

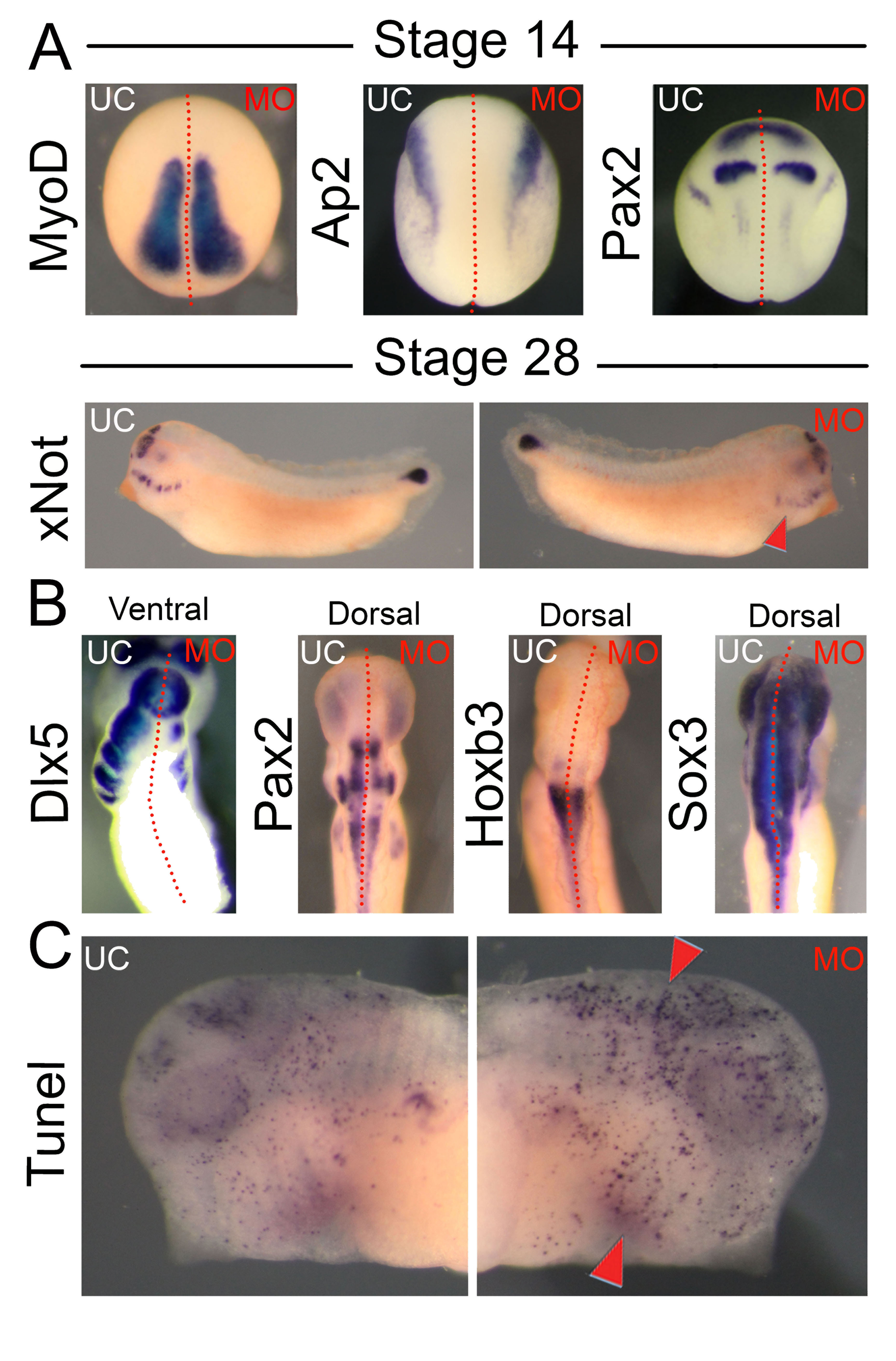

Supplement: S3 Fig — A) Somite, CNC and neural development appear intact in nol11 morphants at stage 14 as assayed by myoD, ap2 and pax2 expression. Expression of xnot appears reduced at stage 28. B) Reduced dlx5 expression and BA hypoplasia on treated side of a stage 28 embryo. Neural patterning is normal at this stage as assayed by expression of pax2, hoxb3 and sox3. C) Whole mount TUNEL staining of treated and untreated sides of a stage 28 embryo. Note the increased staining in the craniofacial regions of the morphant side. (TIF) [file pgen.1005018.s003.tif]

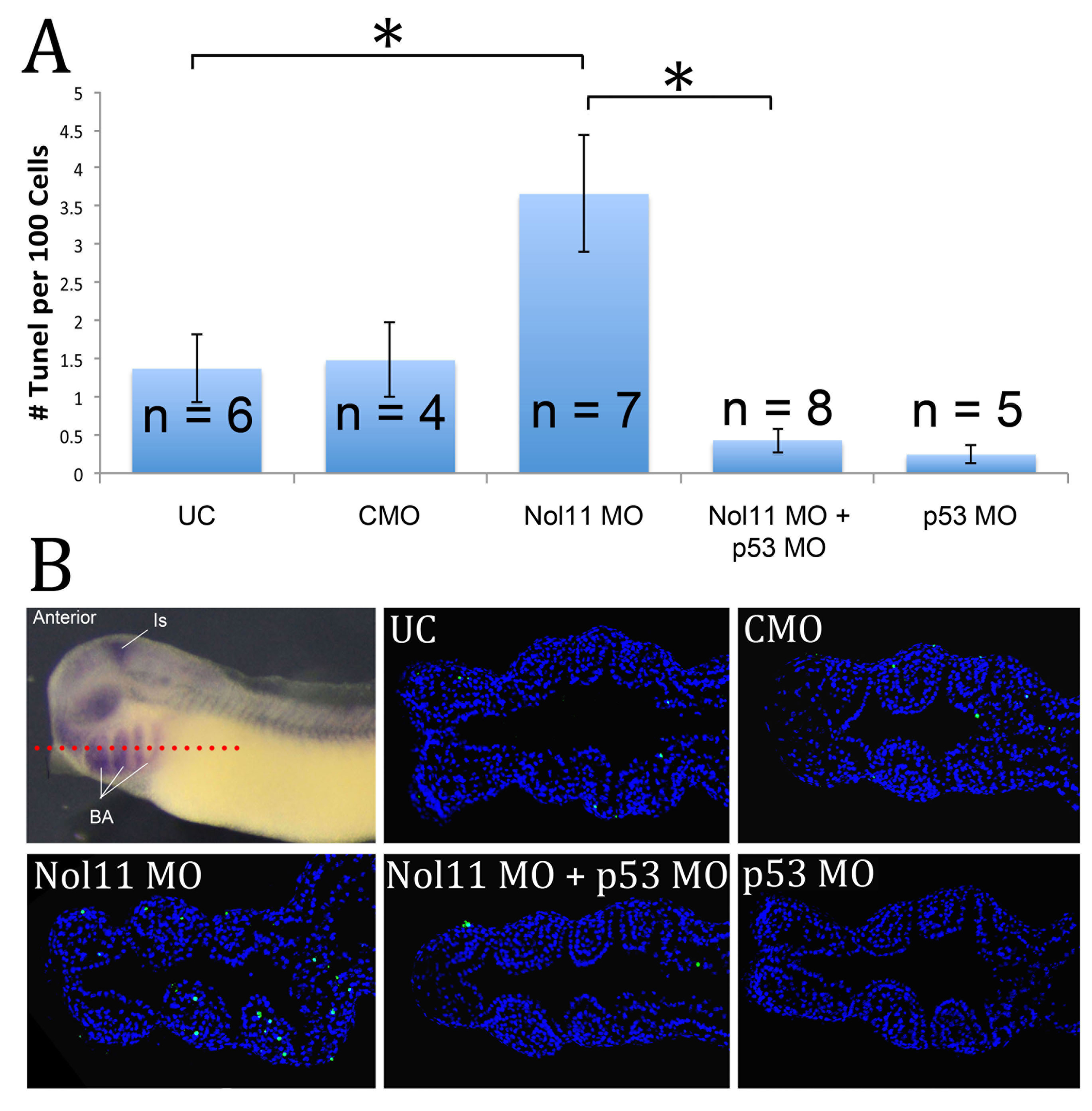

Supplement: S4 Fig — A) Graph of the number of Tunel positive cells per 100 cells present in sections of the branchial arch region of stage 28 UC, CMO, Nol11 MO, Nol11 MO + p53 MO, and p53 MO only injected embryos (* = P < 0.05). B) Whole mount stage 28 embryo with plane of section represented by the red dotted line. Representative Tunel stained (green) sections from each control and morphants. (TIF) [file pgen.1005018.s004.tif]

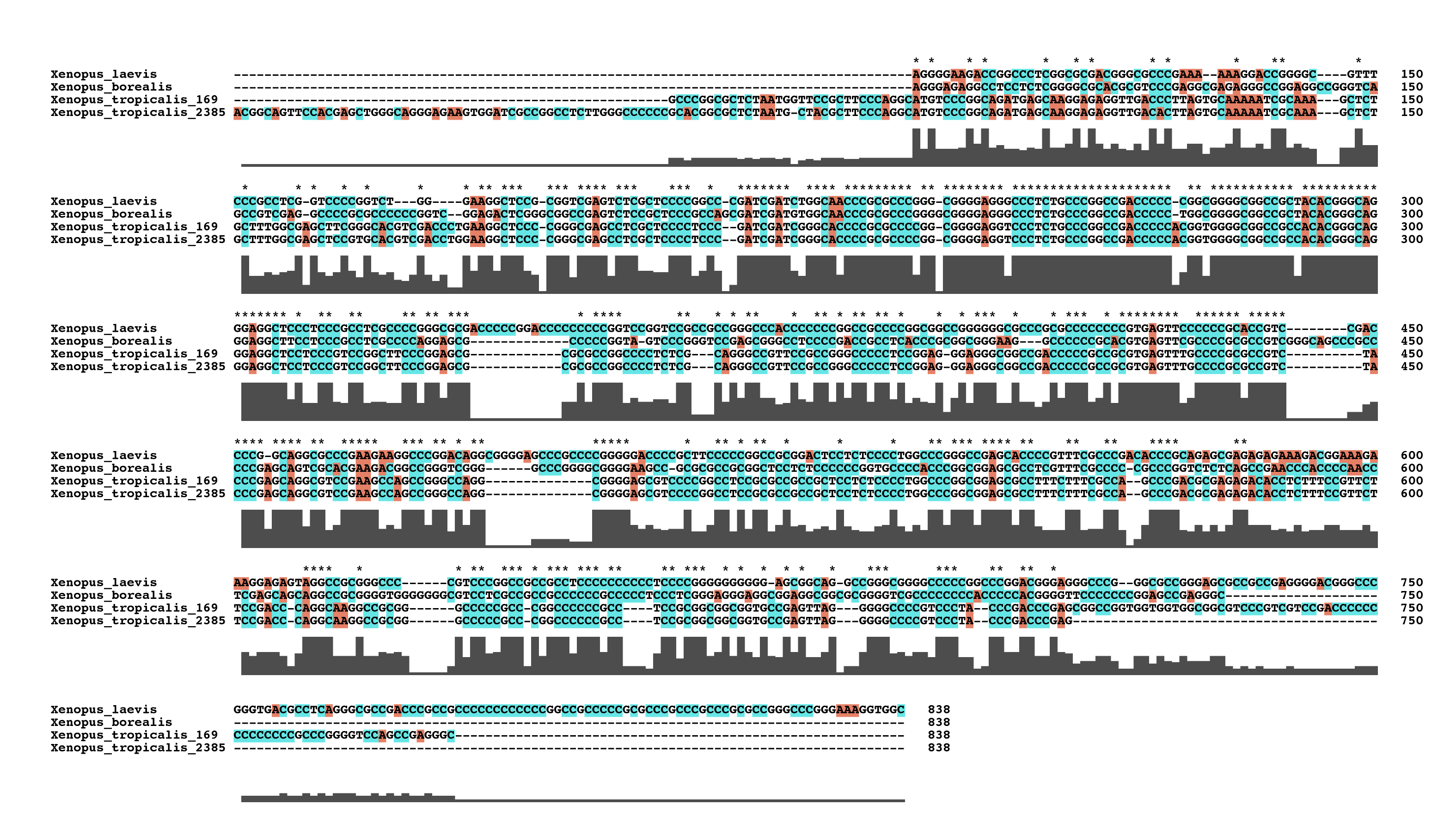

Supplement: S5 Fig — The 5’ETS sequences for X. laevis (GeneBank ID: X02995.1; nucleotides 318–1029) and X. borealis (GeneBank ID: X00184.1; nucleotides 545–1156) were aligned to X. tropicalis scaffolds 169 and 2385 from the Xentr7.1 database. (TIF) [file pgen.1005018.s005.tif]

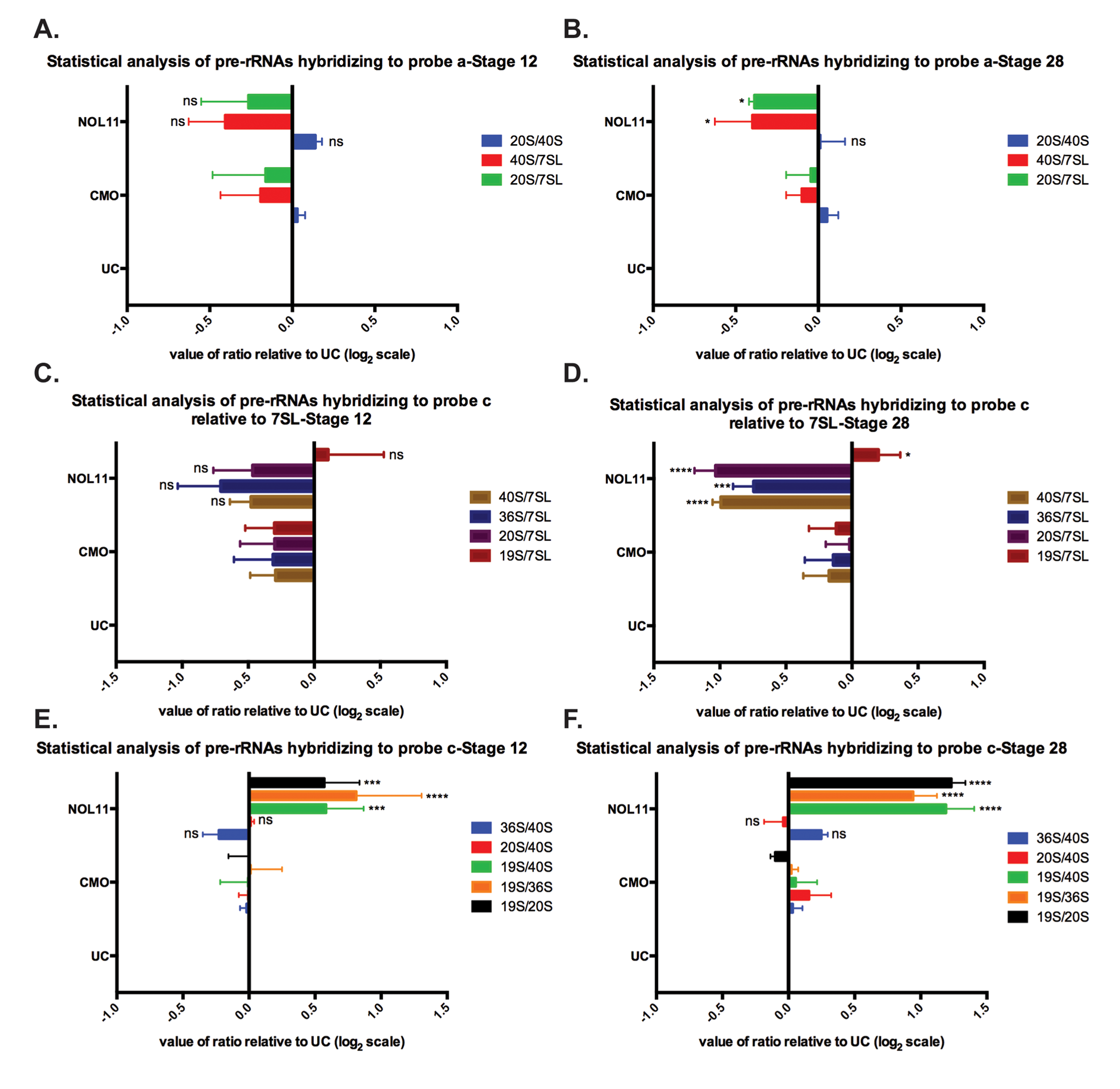

Supplement: S6 Fig — RNAs were quantitated from northern blots using a phosphorimager (Bio-Rad Personal Molecular Imager). All ratios are representative of three biological replicates (n = 3). The levels of all RNAs were normalized to the uninjected control (UC). All statistical analyses for significance for Nol11-depleted embryos (NOL11) were performed compared to the control morpholino injected embryos (CMO). A. Pre-rRNA levels in Nol11-depleted embryos are not significantly affected at stage 12 compared to CMO as shown by a probe in the 5’ETS (probe a). B. At stage 28, the levels of 40S and 20S pre-rRNAs, are significantly decreased relative to the loading control 7SL RNA for Nol11-depleted embryos compared to CMO as shown by probe a. This is consistent with decreased pre-rRNA transcription. C. Pre-rRNA levels relative to the 7SL RNA for Nol11-depleted embryos are not significantly affected compared to CMO at stage 12 as shown by a probe in the ITS1 (probe c). D. At stage 28 for Nol11-depleted embryos, the 40S, 36S, and 20S pre-rRNAs are all significantly decreased relative to the 7SL RNA compared to CMO as shown by probe c. This is consistent with decreased pre-rRNA transcription. However, the levels of 19S are significantly increased relative to the 7SL RNA compared to CMO as this precursor accumulates in Nol11-depleted embryos. This is indicative of a pre-rRNA processing defect. E. and F. For both stage 12 and 28 Nol11-depleted embryos, the ratios of 19S/40S, 19S/36S, and 19S/20S are significantly increased compared to CMO indicating accumulation of the 19S pre-rRNA relative to the other three pre-rRNAs that hybridize with probe c. This is indicative of a pre-rRNA processing defect. [ns = p>0.05 (not significant), * = p≤0.05, ** = p≤0.01, *** = p≤0.001, **** = p≤0.0001] (TIF) [file pgen.1005018.s006.tif]
